# Understanding the impact of sociosexual interactions on sleep using *Drosophila melanogaster* as a model organism

**DOI:** 10.3389/fphys.2023.1220140

**Published:** 2023-08-21

**Authors:** Sukriti Mishra, Nisha Sharma, Shahnaz Rahman Lone

**Affiliations:** Department of Zoology, Central University of Punjab, Bathinda, Punjab, India

**Keywords:** sleep, courtship, sexual interaction, *Drosophila*, after effects

## Abstract

Sleep is conserved across species, and it is believed that a fixed amount of sleep is needed for normal neurobiological functions. Sleep rebound follows sleep deprivation; however, continuous sleep deprivation for longer durations is believed to be detrimental to the animal’s wellbeing. Under some physiologically demanding situations, such as migration in birds, the birth of new offspring in cetaceans, and sexual interactions in pectoral sandpipers, animals are known to forgo sleep. The mechanisms by which animals forgo sleep without having any obvious negative impact on the proper functioning of their neurobiological processes are yet unknown. Therefore, a simple assay is needed to study how animals forgo sleep. The assay should be ecologically relevant so it can offer insights into the physiology of the organisms. Equally important is that the organism should be genetically amenable, which helps in understanding the cellular and molecular processes that govern such behaviors. This paper presents a simple method of sociosexual interaction to understand the process by which animals forgo sleep. In the case of *Drosophila melanogaster*, when males and females are in proximity, they are highly active and lose a significant amount of sleep. In addition, there is no sleep rebound afterward, and instead, males engaged in sexual interactions continue to show normal sleep. Thus, sexual drive in the fruit flies is a robust assay to understand the underlying mechanism by which animals forgo sleep.

## Introduction

### Protocol

Sleep is highly conserved and found across different taxa; however, the purpose of sleep is debatable. Animals such as elephants and horses sleep for a few hours, while some bat species sleep for up to 20 h (Allada and Siegel, 2008). Despite being vulnerable to predators during sleep, animals still undergo this complex behavior, suggesting the importance of sleep. Sleep plays a crucial role in human health, and sleep problems have great significance in modern-day living. In developed countries, insomnia has become a pervasive problem; for example, one-third of the population of the United States suffers from it (Roth, 2005). Insomnia leads to learning and memory problems and is associated with neuropsychiatric disorders such as schizophrenia, bipolar disorder, and depression (Roth, 2005). In addition, shift work strongly affects sleep and causes various health problems (Lack and Wright, 2007; Jehan et al., 2017). Some studies suggest that sleep has restorative functions (Cirelli and Tononi, 2008; Tononi and Cirelli, 2014). An alternative explanation for sleep is adaptive inactivity to conserve energy (Siegel, 2009), which challenges the view about a fixed amount of sleep for optimal performance.

Sleep is incompatible with many behaviors. Male pectoral sandpipers are polygynous and spend much of their time defending territories and chasing females (Lesku et al., 2012). Despite spending more time in male-male competition for the females in the mating season and sleeping less, they maintain high neurobehavioral performance. Interestingly, males who sleep the least can father more offspring (Lesku et al., 2012). A diverse array of migratory birds engage in nonstop flight, which continues for days and weeks, and, hence, the amount of sleep is reduced manifold, whereas part of compensatory sleep is performed on the wing. For example, frigate birds sleep less than an hour—7.4% of the sleep they perform on land—Suggesting the importance of attention during flight (Basner et al., 2013; Rattenborg et al., 2016). In the case of cetaceans, after birth, the mother and calves sleep for only 5–6 months, which probably helps them escape predation and helps in their development (Lyamin et al., 2005). Thus, in many ecological situations, the animals forgo sleep to perform the most pressing functions.

In 1982, Borbely (Borbély, 1982) proposed a two-component model that suggests the involvement of circadian and homeostatic components of sleep. This model advocates that the circadian oscillator sets the onset and offset of sleep, while the homeostatic oscillator tracks the sleep and compensates for recent sleep losses. Sleep in fruit flies has many correlates with mammalian sleep (Hendricks et al., 2000; Shaw et al., 2000; Tononi and Cirelli, 2014). In addition to these well-recognized factors, sleep is also modulated by age, sex, diet, and several environmental conditions in flies (Keene et al., 2010; Linford et al., 2012; Lone and Sharma, 2012) When an organism ages, the sleep architecture changes and becomes more fragmented (Li et al., 2018). In *Drosophila*, the most significant changes start after middle age (Koh et al., 2006). Starvation leads to food-searching behavior in the flies, which increases activity and decreases sleep (Keene et al., 2010) and is regulated by the adipokinetic hormone (AKHR) receptor, the insect analog of mammalian glucagon. Silencing octopaminergic neurons that express these receptor neurons prevents starvation-driven hyperactivity (Yu et al., 2016). Thus, sleep in mammals and flies is modulated by a whole range of common factors.

Sleep in fruit flies is defined as 5 min of inactivity and is usually recorded by the *Drosophila* Activity Monitor (DAM). Two pairs of emitters and receivers are placed to make a pair of cross beams and, when a fly crosses the middle of the tube, it is recorded as a single activity bout. Inactivity of 5 min is counted as one sleep bout in *D. melanogaster* (Hendricks et al., 2000; Shaw et al., 2000). Social interactions, including sexual interactions, are crucial for sleep (Ganguly-Fitzgerald et al., 2006; Fujii et al., 2007; Lone and Sharma, 2012; Lone et al., 2016). The neuronal circuit underlying courtship is well studied (Hall, 1994; Dickson, 2008; Yamamoto and Koganezawa, 2013), but how sexual cues impact sleep is still unclear. When in proximity of each other, males and females show increased activity (Fujii et al., 2007), resulting in sleep loss (Lone and Sharma, 2012). Central to male courtship are genes and neurocircuitry that have been extensively studied (Villella and Hall, 2008) and involve a series of events programmed by two zinc-finger transcription factors, *fruitless* (*fru*) and *doublesex* (*dsx*) (Manoli et al., 2006). Later studies have shown that sleep loss is controlled by the group of FRU-positive P1 neurons. Other studies have identified the importance of the male-specific octopamine neurons, which reduce female-induced sleep loss in males when silenced (Machado et al., 2017). Further, studies have suggested the importance of olfaction and other sensory stimuli in this behavior (Lone and Sharma, 2012; Beckwith et al., 2017).

The current study is focused on a reliable and straightforward assay, that is, vital in understanding the relationship between sex drive and sleep. In the current protocol, after recording 1 day of baseline sleep, sociosexual interactions were restricted for 1 day and the flies were observed for another day to observe the after-effects, if any. These studies suggest that sleep is severely compromised during sociosexual interactions.

#### 1 Preparation of corn food

1.1 To prepare 1 L of corn food, heat 500 mL of water until lukewarm (approximately 40°C, as confirmed with a thermometer), and add 12 g of agar as a solidifying agent. Boil the mixture thoroughly with continuous stirring until the agar dissolves completely in water.

1.2 In another container, mix 100 g of corn powder, 40 g of sugar, and 45 g of yeast with 500 mL of water using a blender to prevent the formation of lumps.

1.3 Pour the mixture from Step 1.2 into the agar solution prepared in Step 1.1. Bring the mixture to a boil and let it boil for a few minutes before removing it from heat.

1.4 Dissolve 1 g of benzoic acid completely in 10 mL of ethanol to prepare food preservatives for 1 L of food. Adjust the amount of preservatives according to the food requirements.

1.5 Let the food cool down, checking the temperature with a thermometer, and add the preservatives from Step 1.4 when the temperature reaches 60°C.

1.6 Mix 10 mL of propionic acid thoroughly into the food.

1.7 Pour the food into vials (25 mm × 95 mm), let it cool down, and seal with cotton plugs. Use these food vials to culture the flies.

1.8 Add four young females and three males (up to 10 days old) to each food vial and create multiple vials using this combination. Wait for the flies to emerge after 9–10 days if raising the cultures at 25°C.

#### 2 Preparation of the locomotor tubes

2.1 To prepare 5% sucrose, dissolve 12 g of agar in 1 L of distilled water. Weigh 50 g of sucrose, add to the agar-water mixture, and heat it until it boils.

2.2 Allow the 5% sucrose to cool down, continuously checking it with a thermometer, and, at 60°C, add the preservative prepared by dissolving 1 g of benzoic acid in 10 mL of ethanol per 1 L of sucrose food.

2.3 Pour this liquid sucrose medium into Petri plates (15 mm × 90 mm) and let it cool. Meanwhile, make a tube bunch by packing 30–40 tubes (5 mm × 65 mm, diameter × length) tied together with a rubber band. Adjust the number of bunches according to the experimental need.

2.4 When the food cools slightly and is still liquid, gently place these bunches vertically in the Petri plates and allow the hot sucrose medium to enter inside tubes by capillary action.

2.5 When the food solidifies, take out the bunches from the food and clean them using tissue or wipes.

2.6 Melt paraffin wax in the bowl of the wax heater and dip the tubes into the hot wax to seal the end of the tube in which food is present.

#### 3 Preparation of the flies for the experiments

3.1 Collect freshly eclosed flies, sex them, and keep them in 12 groups (six for each sex) of 20–30 flies.

3.2 Maintain these flies under a 12:12 h light-dark (500:0 Lux) cycle with 70% humidity in an incubator set at a constant temperature of 25°C.

3.3 Age these flies until they become 4–5 days old.

#### 4 Experimental setup

4.1 Preparation of the incubator and installation of DAM software on the storage desktop.

4.1.1 Prepare the incubator in advance by setting the optimum temperature (25°C ± 0.05°C) and the timing of the light-dark cycle (12:12 h).

4.1.2 Download the DAM system software from the Table of Materials and install it on a storage desktop computer.

4.1.3 Open the DAM software and set the reading interval to 1 min by clicking on “Preferences” and changing the setting.

4.1.4 Set the range of the loaded DAM monitors in “Monitor Numbers”. When the signals turn from red to green, the monitors are connected to the USB interface. If the DAM system fails to recognize the monitors, refer to the DAM software details for troubleshooting.

##### 4.2 Loading of flies into activity tubes for sleep recording

4.2.1 Anesthetize 4–5-day-old flies with carbon dioxide and load them as single males and females into an activity tube (5 mm × 65 mm) for recording. Adjust the number of males and females according to the experimental requirements.

4.2.2 Load six groups of single flies, including naïve males and females that do not experience social interaction (NM and NF), males and females that interact with same-sex flies (MM and FF), and males and females that experience sociosexual interactions (MF).

4.2.3 Place the activity tubes in *Drosophila* Activity Monitors (DAM2) to record any locomotion of the flies as they cross the middle of the tube.

4.2.4 Place the monitors in the incubators, connect them with the data collection system via telephone wires provided by the manufacturer, and check the connection on the desktop computer on which the DAM scanning software is loaded.

4.2.5 Exclude data from the loading day and the next day to avoid any potential impact of the anesthesia. Record the baseline sleep of individual males and females for 1 day.

4.2.6 On the second day, join the ends of the locomotor tubes at ZT0 to bring together single individuals. Look for social groups formed when a fly joins another: MM, FF, and MF. Keep NM and NF as isolates through the experiment. (Note: ZT0 is lights on and ZT12 is lights off.)

4.2.7 After 24 h of social interaction, separate the flies at ZT0 by joining the open end of an empty tube to the tube carrying two flies and waiting for one of them to move into the empty tube. Keep the tubes carrying the males and females in their respective DAM monitors.

4.2.8 Shift the DAM monitors from the incubator to the part of the laboratory with light and temperature conditions similar to the incubator to mix and separate flies, as described in Step 4.2.6 and Step 4.2.7. Ensure that the DAM monitors remain connected to the DAM system during these steps.

4.2.9 Continue to record the flies for another day after separation. Compare the after-effects of the sexual drive on sleep by using controls that were not subjected to the sexual interaction (naïve groups) and same-sex males and females.

#### 5 Data collection and analysis

5.1 Process the data collected in 1 min bins using the downloaded DAM File scan for further analysis.

5.2 Save data from day 1—Lights on (if 8 am is lights on, save data from 8:01 day 1).

5.3 Analyze the sleep data using the Sleep and Circadian Analysis MATLAB Program (SCAMP) (Donelson et al., 2012), which is freely available on the manufacturer’s website, using 5 min of inactivity as the threshold for a sleep bout (Hendricks et al., 2000; Shaw et al., 2000).

5.4 Dump downloaded SCAMP software and 1 min channels files into the folder and name it *sleep data*.

5.5 Open *Matlab* and set the path for *sleep data* folder by choosing the *set path under the file option*. Choose *add all the sub-folders.*

5.6 Type *scamp* in the command window of Matlab. Wait for a window to pop up, and choose the folder containing 1 min channel files.

5.7 On the left-hand side, select the *monitor* and channel files associated with the monitor that appears with corresponding editable text boxes, where one can specify the name of the group related to a specific monitor. Name the first channel as *group 1* and click *apply channel 1 group name to entire board.*

5.8 Repeat the exercise for the 2nd and 3rd groups.

5.9 Select the monitors belonging to groups 1, 2, and 3 and click *load individual sleep plots.*

5.10 Uncheck the *channels containing dead flies*.

5.11 Click *analyze selected data*, and wait for a window to pop up. On the left-hand side, check boxes *s30-min of sleep/30 min* and *stdur-total sleep duration* **.** Select other parameters if needed.

5.12 On the right-hand side, under select groups to analyze, check groups 1, 2, and 3. Under select days to average, check Day 1. Under select all groups, check graph data for visualization and export data.

5.13 Click average selected days, graph selected days, and export all data.

5.14 In the data folder, export the data and open the *BinAVGs30. csv* file in the spreadsheet, average across the flies for sleep profile, and use this information in step 6.1.

5.15 Use “*stdur-total sleep duration*” for further analysis in Step 6.2.

5.16 Repeat this exercise for the second and third days.

#### 6 Plotting activity and sleep data

##### 6.1 Plot sleep profile

6.1.1 Open the graph pad and select *XY*, where X is number, and click on -Y (plot error values already calculated elsewhere) and then select *mean and sem*.

6.1.2 Create the graph; copy the data from the spreadsheet to groups A–C.

6.1.3 Now, click on *insert graph* from existing data, choose *XY graph* | *plot points* | *connecting lines with error bar.*

##### 6.2 Plot nested plots

6.2.1 Plot total sleep in *nested plots*.

6.2.2 Choose *column* and click on *enter replicates value, stacked in columns*.

6.2.3 Copy the calculated total sleep values from the spreadsheet and paste them into *groups A–F.*

6.2.4 Click on *insert graph* from existing data and choose the *column to scatter plot* with mean and SEM.

6.2.5 Change the color code based on preference by clicking on the data set.

6.2.6 Choose the color and shape of the symbol of each data set from the *format graph* table.

6.2.7 Click on *apply.*

#### 7 Statistical analysis

7.1 As the data obtained do not follow the normal distribution, perform the Mann Whitney *U*-test. Adjust the *p*-value for multiple comparisons to rule out false positives due to type 1 error. Consider *p* < 0.05 as the statistical significance level.

7.2 Prepare the figures: sleep profiles, sleep amount, and sleep loss.

## Representative results

**FIGURE 1:**
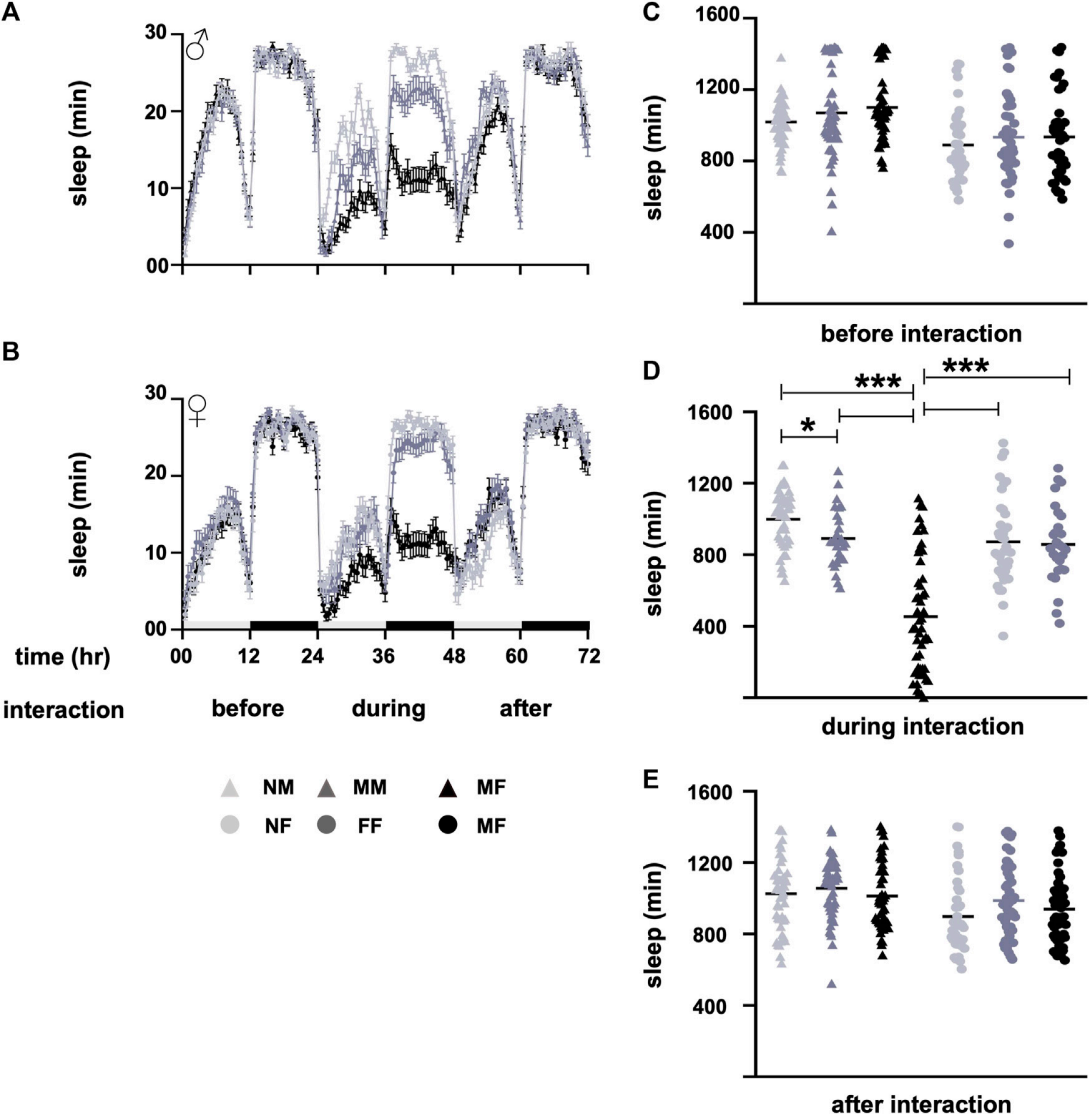
Sleep profiles before, during, and after interactions. **(A)** Sleep profile of males (NM, MM, and MF), and **(B)** sleep profile of females (NF, FF, and MF) before, during, and after the interaction. Sleep waveforms represent means with errors as standard error of the mean. **(C)** Sleep of different groups before interaction, **(D)** during interaction, and **(E)** after interaction. Before the interaction, the sleep of male and female groups is similar (*p* > 0.5). During the interaction, the sleep of the MF group is lower (*p* < 0.0001) than the sleep of NM, NF, MM, and FF. The sleep of MM is lower (*p* = 0.04) than the sleep of NM. Sleep is restored to normal after the interaction, and the sleep of different male and female groups is similar (*p* > 0.05). The light and dark bars in panel B along the x-axis of the sleep profiles denote daytime and nighttime sleep. The horizontal lines in the nested dot blots **(D)** represent the means. Light grey represents NM and NF, dark grey represents MM and FF, and black represents MF in both the sleep profile and bar graphs. The triangles represent males, and the circles represent females in the sleep profiles. During interaction, MF are represented by the black triangles. Sample size “n” varies from 28 to 53 for different groups. *p < 0.05, and ***p < 0.0005. Abbreviations: NM, naïve males; MM, male-male; MF, male-female; NF, naïve females; FF, female-female; MF, male-female.

Among the different male and female fly groups, the naïve male and female groups, NM and NF, did not experience interaction. However, the same-sex groups, MM and FF, and opposite-sex groups, MF and FM, underwent interaction with either the same or the opposite sex. The data were tested for normal distribution using the Shapiro-Wilk test. Non-parametric data were compared by the Mann-Whitney *U*-test, and the *p-*value was adjusted for multiple comparisons. Prior to interaction, the sleep of different male and female groups was similar (*p* < 0.05, Figures 1A–C). During interaction for a period of 24 h, the sleep of MM was lower than that of NM (*p* = 0.04, Figures 1A, D), whereas the sleep of MF was lower (*p* < 0.0001, Figures 1A, D) than the sleep of MM and NM. The sleep of naïve females NF was similar (*p* = 0.92, Figures 1B, D) to the sleep of FF, whereas the sleep of MF was lower (*p* < 0.0001, Figures 1B, D) than the sleep of both NF and FF. After the interaction, flies were separated without anesthesia as mentioned above; sleep was similar (*p* < 0.05, Figures 1A, B, E among the male and female groups.

## Discussion

In the current protocol, both males and females were housed together for 24 h to investigate the impact of sexual interactions on sleep. During male-female interactions, sleep loss was observed, with males taking the initiative and playing an active role in these interactions (Lone and Sharma, 2012). One drawback of this protocol is the inability to accurately assess the sleep of either partner during the interaction. However, despite this limitation, when we compared mixed-sex male-female social groups with same-sex social groups (male-male and female-female), the sleep duration of mixed-sex flies was found to be lower than that of same-sex flies, as well as naive males and females. This protocol provides an easily analyzable method for molecular and cellular screening to understand physiological mechanisms involved in socio-sexual interactions. Alternatively, video analysis of social interactions can be utilized to assess the sleep of individual flies, providing a more precise measurement of the sleep (Donelson et al., 2012; Gilestro, 2012). However, it should be noted that this method is time-consuming and may require extensive efforts for analysis.

The transfer of the sex peptide from the males to the female is believed to be responsible for daytime sleep loss in mated flies, and this sleep loss is found across strains and is unaffected by food composition (Isaac et al., 2010). These results are contrary to the findings presented here that female sleep returns to normal after the sociosexual experience. The discrepancy in the results may be due to differences in the group size and the arena of social interaction (Isaac et al., 2010; Garbe et al., 2016). According to previous research by Hendricks et al., in 2000, sleep rebound in flies occurs for a few hours immediately after sleep loss and can still be observed up to 2 days after sleep deprivation. However, when we analyzed our data for recovery sleep over a 2-day period following sleep deprivation due to social interactions, we found no evidence of any recovery sleep. It is worth noting that sleep recovery tends to be more prominent when the duration of sleep deprivation is extended to 12–24 h, rather than just 12 h of deprivation during the night. This indicates that the duration of sleep deprivation plays a significant role in the extent of sleep rebound.

Many studies have utilized the described paradigm to successfully investigate the various molecules involved in socio-sexual interactions. Fijii et al. (2007) initially demonstrated that locomotor activity increases when males and females are in close proximity. In 2012, we adopted the activity tubes as a means to record sex drive and discovered that it not only affects activity levels but also impacts both day and night sleep (Lone and Sharma, 2012). In insects, including *Drosophila melanogaster*, there are 45 different olfactory receptor neurons (ORNs) located in the antennae and maxillary palp (van Naters and Carlson, 2007; Masse et al., 2009). Among these, the ORNs expressing the co-receptor *Or47b* play a vital role in these interactions (Lone and Sharma, 2012). Non-volatile pheromones are detected by the distal tips of the males’ forelegs, housing the degenerin/epithelial sodium channel (DEG/ENaC) family receptors. Mutations in these receptors, such as Ppk25, Ppk23, and Ppk29, have been shown to lead to reduced courtship behavior (Liu et al., 2012; Starostina et al., 2012; Toda et al., 2012; Vijayan et al., 2014). Notably, mutations in Ppk23 result in the suppression of sleep loss during socio-sexual interactions (Beckwith et al., 2017). Subsequent studies have identified a cluster of octopaminergic neurons that do not express FRU but are interconnected with Fru-positive neurons (Chen et al., 2017; Machado et al., 2017). These octopaminergic neurons contribute to sexual interactions and modulate sleep patterns.

We propose that sleep deprivation in male *Drosophila* may have a similar impact on fitness, as reported by Lesku et al. (2012). Specifically, sleep-deprived males may be capable of copulating with a large number of virgin females. Interestingly, certain animals, such as birds and cetaceans, can forego sleep loss when engaging in behaviors crucial for their survival (Lyamin et al., 2005; Rattenborg, 2006; Basner et al., 2013). These examples demonstrate that certain animals can undergo sleep deprivation without adverse effects on their physiology, ultimately improving their overall inclusive fitness. In conclusion, this straightforward and reliable assay provides a valuable tool for comprehending the molecules and neurons underlying sleep loss during sex drive.

## Data Availability

The original contributions presented in the study are included in the article/Supplementary Material, further inquiries can be directed to the corresponding author.
